# Psychological Distress and Associated Factors Among High-School Students in Makkah, Saudi Arabia: A Cross-Sectional Study Using the Arabic GHQ-30

**DOI:** 10.3390/ijerph23060733

**Published:** 2026-05-30

**Authors:** Arwa Hesham Hashim, Adeel Ahmed Khan, Aalia Akhtar Hayat

**Affiliations:** 1 Saudi Board Program of Preventive Medicine, Program, Makkah Health Cluster, Makkah 24242, Saudi Arabia; aaahmedkhan@moh.gov.sa; 2 Department of Child Psychiatry, Maternity and Children Hospital, Makkah Health Cluster, Makkah 24242, Saudi Arabia

**Keywords:** adolescent mental health, psychological distress, GHQ-30, school-based screening, high-school students, Saudi Arabia, bullying, sleep duration, physical activity, cross-sectional study

## Abstract

**Highlights:**

**Public health relevance—How does this work relate to a public health issue?**

- Adolescent mental health problems often begin during the school years, yet many affected students remain unidentified until symptoms become more severe.
- This study addresses a school-based public health issue by estimating the prevalence of psychological distress among high-school students in Makkah and examining associated demographic, socioeconomic, and behavioral factors.

**Public health significance—Why is this work of significance to public health?**

- The study found a high prevalence of psychological distress in this population, highlighting the need for earlier identification and support for adolescents at risk.
- Bullying, female gender, insufficient sleep, and lower physical activity were significant correlates of distress and related symptom clusters, indicating modifiable targets for prevention strategies.

**Public health implications—What are the key implications or messages for practitioners, policy makers and/or researchers in public health?**

- School-based mental health screening using brief validated tools such as the Arabic GHQ-30 may help identify vulnerable students and support timely referral and intervention.
- The findings may inform school health programs and public health policies aimed at strengthening adolescent mental health promotion, bullying prevention, and early supportive services.

**Abstract:**

Background: Mental health problems often begin in adolescence, yet early detection and intervention remain limited. This study assesses the prevalence of psychological distress and its correlates among high-school students in Makkah, Saudi Arabia, and explores whether specific symptom clusters of depression, anxiety and bipolar/mania can be identified using the General Health Questionnaire-30 (GHQ-30). Methods: A cross-sectional survey was conducted during the 2025–2026 academic year using stratified cluster sampling. A total of 535 students aged 15–18 years completed a questionnaire containing the validated Arabic GHQ-30 and demographic, socioeconomic and lifestyle items. The GHQ-30 was scored with binary 0-0-1-1 scoring (cut-off ≥ 6) to define cases of psychological distress. Item clusters were used to screen for probable depression, anxiety and bipolar/mania. Descriptive statistics characterized the sample. Associations were examined using chi-square tests and multivariable logistic regression. Results: Overall, 70.5% of participants screened positive for psychological distress. The prevalences of probable depression, anxiety and bipolar/mania were 33.1%, 28.2% and 31.2%, respectively. In adjusted models, female gender, insufficient sleep, lack of physical activity and exposure to bullying were associated with increased odds of psychological distress; longer sleep was protective. History of mental health conditions was a strong predictor of probable depression, whereas medication use was protective. Older age and higher paternal education were protective for anxiety. Bullying was the most consistent predictor across all symptom clusters. Conclusions: Psychological distress is highly prevalent among Makkah high-school students. Key determinants include gender, sleep duration, lack of physical activity and bullying. Routine school-based mental health screening, sleep-hygiene education, anti-bullying initiatives and early referral pathways are warranted. Further research should examine and validate GHQ-30 item clusters for specific disorders.

## 1. Introduction

Adolescent mental health has become a critical public health priority worldwide. According to the World Health Organization (WHO), around one in seven adolescents experiences a mental health disorder and suicide is among the leading causes of death for this age group [1]. Untreated mental health conditions in adolescence can continue into adulthood, underscoring the need for early detection [1]. Early identification and intervention may mitigate long-term negative consequences, including substance misuse, social withdrawal and academic underachievement [2]. Adolescence is a formative developmental period in which rapid physical, emotional and social changes—including exposure to poverty, abuse or violence—heighten vulnerability to psychological problems [1].

Most mental health disorders emerge in adolescence; nearly half of all disorders begin by age 14 and many cases go unrecognized until adulthood [2]. Because serious psychiatric conditions often start before adulthood, focusing on high-school students (15–18 years) allows screening and intervention during a critical developmental stage [1].

Screening instruments such as the General Health Questionnaire (GHQ) and the Strengths and Difficulties Questionnaire (SDQ) are widely used to identify psychological distress in non-clinical adolescent populations [3,4]. The GHQ has also shown acceptable validity and psychometric performance in adolescent populations [5,6]. The GHQ-30, in particular, demonstrates high reliability and sensitivity across diverse settings [7,8]. It has been validated in Arabic-speaking populations and shows excellent internal consistency and appropriate thresholds when scored using the binary method [9]. In this study, psychological distress is defined using the GHQ-30: respondents scoring six or more on the binary-scored GHQ-30 are considered to screen positive for distress.

Existing regional evidence shows high levels of depressive, anxiety, and stress symptoms among Arab adolescents, with prevalence varying across settings and with socioeconomic disadvantage associated with worse outcomes [10,11]. Saudi-specific studies report comparable rates. For instance, AlYousefi and colleagues identified depression risk factors such as poor parent–child communication and social isolation among Riyadh adolescents [12], while Khan and co-authors documented high levels of depression, anxiety and obsessive-compulsive symptoms among secondary students in the Al-Hasa region [13].

To date, no study has applied the Arabic GHQ-30 to high-school students in Makkah city. Cultural norms, socioeconomic variability and educational contexts in Makkah may uniquely shape adolescent distress patterns. This study addresses that gap by estimating the prevalence of psychological distress (GHQ-30 score ≥ 6) and examining demographic, socioeconomic and academic correlates among high-school students in Makkah.

In addition to measuring overall distress, we explored GHQ-30 item-level clusters to screen for probable depression, anxiety and bipolar tendencies. Although the GHQ-30 is not a diagnostic tool, factor analyses suggest that its items group into dimensions such as depression, anxiety and social dysfunction [7,8,14]. Bipolar disorder often manifests during adolescence, with community prevalence estimated at about 1–2% [15,16,17]. By examining item clusters, we aim to identify students who may be at elevated risk for depressive, anxious or manic-like symptoms. These clusters are exploratory and are intended to flag individuals who may require further clinical assessment rather than to diagnose specific disorders [18].

Therefore, this study had two objectives: first, to estimate the prevalence of psychological distress among high-school students in Makkah and identify its demographic, socioeconomic, and school-related correlates; and second, to explore whether predefined GHQ-30 item groupings could be used as exploratory symptom groupings for probable depressive, anxiety, and manic-like symptoms. We hypothesized that psychological distress would be common in this population and would be associated with female sex, shorter sleep duration, lower physical activity, bullying exposure, and socioeconomic disadvantage. We further hypothesized that the exploratory GHQ-30 item groupings would identify non-mutually exclusive symptom patterns with similar psychosocial correlates, while recognizing that these groupings are hypothesis-generating screens rather than validated diagnostic subscales.

## 2. Methodology

### 2.1. Study Design

This cross-sectional survey was conducted among high-school students in Makkah during the 2025–2026 academic year and is reported in accordance with the STROBE guidelines to ensure methodological quality.

### 2.2. Study Population

Eligible participants were students aged 15–18 years who were enrolled in one of the selected high schools and were able to read and write in Arabic. Students who were absent on the scheduled data collection day were excluded, as were those who did not provide assent or whose parent or guardian did not provide consent. Questionnaires with more than 20% missing GHQ items were considered incomplete and were excluded from the analysis.

### 2.3. Sample Size Calculation

The minimum sample size required was calculated using an expected prevalence of 40.1%, based on prior Saudi youth data reporting a high burden of mental disorders [19], with 95% confidence and a 5% margin of error. Applying a design effect of 1.5 for cluster sampling increased this to 553. Consistent with methodological guidance that surveys should recruit 20–30% more subjects to compensate for potential non-response and missing data, an additional 10% allowance was added, yielding a target sample of 609 students. In practice, 535 questionnaires were returned and included in the analysis. This represents an 87.8% response rate, which falls within the anticipated non-response range and preserves sufficient statistical power for the planned analyses.

### 2.4. Sampling Strategy

According to the Ministry of Education, there are about 105 high schools in Makkah (55 for boys and 50 for girls) across public and private sectors [20]. We employed a stratified random cluster sampling strategy to ensure representation across gender and school sector. The sampling frame was stratified into four categories (boys-public, boys-private, girls-public, girls-private). From each stratum, one school was randomly selected. Within each selected school, two class sections from each of grades 10, 11 and 12 were randomly chosen; all students in the selected sections were invited to participate. This two-stage cluster design was chosen to enhance logistical feasibility while preserving representativeness.

### 2.5. Data Collection Tools

1. General Health Questionnaire (GHQ-30):

We used the validated Arabic version of the GHQ-30. This self-administered screening tool comprises 30 items scored using the binary method (0-0-1-1), yielding a total score between 0 and 30 [3]. The Arabic version has demonstrated high internal consistency (Cronbach’s α ≈ 0.93) and test–retest reliability in primary care populations [9]. Permission to use this version was obtained from its original Arabic-language validators. Respondents scoring six or more on the GHQ-30 were considered to screen positive for psychological distress.

2. Demographic and Socioeconomic Questionnaire:

A custom questionnaire captured demographic and socioeconomic data. Its items were informed by factors previously linked to adolescent mental health, including parental education, family income, household size, academic performance, sleep patterns, physical activity and bullying, based on regional studies [21,22,23,24]. The non-GHQ demographic and socioeconomic items underwent expert content review by a consultant child psychiatrist. The full Arabic questionnaire and its English translation are provided in Supplementary File S1.

### 2.6. Data Collection Procedure

Data were collected using paper-based forms during school hours. A trained data collector administered the questionnaires in the classroom; signed parent consent forms were distributed to the selected students a day before the survey and collected at the start of the session. The teacher assisted with classroom management but did not view students’ responses. Sufficient time (~30–40 min) was provided.

To ensure confidentiality, all survey responses were anonymized using unique codes without names or identifiers. Students who wish to receive their results may voluntarily provide their name and contact information on a separate form attached to the questionnaire. This information was stored securely and separately by the Principal Investigator (PI) and used solely to send individual results. It will not be accessible to other researchers or included in data analysis.

All identifiable data were securely destroyed after results were shared. Electronic files were password-protected, and paper records were stored in a locked cabinet for the duration required by institutional policy.

### 2.7. Statistical Analysis

The GHQ-30 can be scored using either the binary 0-0-1-1 “GHQ” method or Likert scoring. The test publisher notes that thresholds depend on the population and context; for the GHQ-30, there is no universal default, and users are advised to select a threshold based on prior research [3,25]. Suggested binary thresholds for the GHQ-30 range from 4/5 to 6/7. A validation study of the Malay GHQ-30 found that a cut-off of 5/6 (≥6) achieved high sensitivity (87.5%) and specificity (80.6%) for detecting psychological distress. Given the expected high prevalence of distress in adolescents and the need to reduce false-positive results, we adopted a threshold of ≥6, consistent with this validation study and with recommendations from the GHQ manual and WHO-related work suggesting that the optimal threshold may lie between 5/6 and 6/7. This threshold has also been used in Arabic-speaking populations and is appropriate for screening purposes [7,9].

Although the GHQ-30 yields an overall distress score, factor-analytic studies have shown that its items cluster into dimensions resembling depression, anxiety, interpersonal dysfunction, and related constructs. For example, a large Japanese population study identified factors labelled depression, anxiety and tension, anergia, interpersonal dysfunction, difficulty in coping, insomnia, anhedonia, and social avoidance. Similarly, factor analyses in student samples have reported components such as general dysphoria, social functioning, depressive thoughts, and insomnia. Building on this evidence, and on the four subscales established for the related GHQ-28 (somatic symptoms, anxiety/insomnia, social dysfunction, and severe depression), we grouped GHQ-30 items into exploratory clusters reflecting depressive, anxious, and bipolar/mania-related symptoms. This clustering was intended to screen for probable symptom patterns rather than to diagnose specific disorders [7,8,14].

Accordingly, GHQ-30 items were grouped into three exploratory symptom clusters. The depression cluster comprised nine items (16, 17, 22, 23, 24, 25, 26, 27, and 29), the anxiety cluster comprised ten items (1, 2, 3, 14, 15, 18, 19, 21, 28, and 30), and the bipolar/mania cluster comprised four items (3, 14, 28, and 30). Cut-offs for positive screens were defined as five or more for depression, six or more for anxiety, and three or more for bipolar/mania. Each cluster score was calculated by summing the binary-scored responses to the corresponding items. These exploratory subscales were used only to identify probable symptom patterns and should not be interpreted as diagnostic measures.

Data were entered and analysed using IBM SPSS Statistics for Windows, version 28.0 (IBM Corp., Armonk, NY, USA). Completeness checks were performed before analysis. Descriptive statistics were used to summarise participants’ sociodemographic and behavioural characteristics. Categorical variables were expressed as frequencies and percentages, whereas continuous variables were summarised using mean ± standard deviation or median and interquartile range, depending on their distribution. GHQ-30 items were scored using the binary 0-0-1-1 method, and total and cluster scores were calculated accordingly; cases of psychological distress were defined by a GHQ-30 score of ≥6. Normality of continuous variables was assessed using the Shapiro–Wilk test, and homogeneity of variances was assessed using Levene’s test; non-parametric alternatives were used when assumptions were violated. Associations between categorical variables were examined using chi-square tests. Variables with *p* < 0.05 in bivariate analyses were entered into multivariable binary logistic regression models to identify independent predictors of overall psychological distress and each symptom cluster (depression, anxiety, and bipolar/mania), with results reported as adjusted odds ratios (aORs) and 95% confidence intervals. All candidate predictors were entered simultaneously using the enter method in SPSS (Analyze → Regression → Binary Logistic). Because the SPSS output did not include covariance parameters or intraclass correlation coefficients, no random effects were specified in the final models, and the analyses therefore assumed independent observations. Statistical significance was set at *p* < 0.05. Because the study used a stratified two-stage cluster sampling design, a complex-samples approach would have been preferable for variance estimation. However, due to the available analytic setup, the final regression analyses were conducted using standard logistic regression. Accordingly, the resulting standard errors, confidence intervals, and *p*-values should be interpreted with caution, as clustering-related variation may not have been fully accounted for.

### 2.8. Ethical Considerations

Ethical approval for this study was obtained from the Institutional Review Board of the Ministry of Health, Makkah (IRB No. H-02-K-076-0725-1383, issued 27 July 2025). Approval to conduct the survey in schools was also secured from the appropriate educational authorities and from the principals of all participating schools.

Permission to use the Arabic GHQ-30 was obtained by email from its original Arabic-language validator, Dr. Gamal Abdel-Rasoul. Written informed consent was obtained from parents or legal guardians, and assent was obtained from all participating students. Participation was entirely voluntary, and students could withdraw at any time without consequence. To protect confidentiality, all data were de-identified, securely stored and accessible only to the principal investigator. Students who voluntarily provided their names and contact information to receive their results had that information stored separately and used solely for that purpose. The principal investigator communicated the GHQ-30 findings to each student in a sensitive manner, along with advice to seek counselling if the score suggested distress.

## 3. Results

### 3.1. Sample Characteristics

The survey included 535 high-school students from Makkah. The median age was 17 years; 45.8% were 17-year-olds, 24.3% were 16 years, 20.4% were 18 years and 9.5% were 15 years. Male students comprised 67.9% of the sample. More than half of the respondents were in the third secondary-school year (51.4%), 30.7% were in the second year and 17.9% were in the first year. Most students attended government schools (68.4%), while 31.6% attended private schools. Only 1.1% reported a history of psychological or mental conditions and 0.7% were taking psychiatric or neurological medications. Nearly half of respondents preferred not to disclose their monthly household income; among those who answered, 16.6% reported incomes above 15,000 Saudi rials and 12.5% reported incomes below 5000 rials. University education was the most common highest educational attainment among fathers (39.8%) and mothers (44.1%). Family size varied: 41.9% had three to four siblings and 39.4% had five or more. These characteristics are detailed in Table 1.

### 3.2. Lifestyle Habits and Experiences

Table 2 summarizes students’ lifestyle habits. Almost half (45.2%) reported sleeping 4–6 h per night; 37.6% achieved the recommended 7–9 h and 11% slept more than 9 h. Physical activity levels were generally low: 32.7% reported no weekly physical activity and 26.7% engaged in less than 60 min per week; only 11% exceeded 400 min of activity per week. Most students (77.6%) had not been subjected to bullying in the past three years; 20.4% reported verbal or psychological bullying and 1.3% reported combined physical and psychological bullying. Academic performance was high overall (mean score 94.5 ± 8.0).

### 3.3. Prevalence of Psychological Distress and Symptom Clusters

The General Health Questionnaire-30 (GHQ-30) was scored using the binary 0-0-1-1 method. A total GHQ-30 score ≥ 6 defined psychological distress. Overall, 70.5% of students screened positive for psychological distress. Item-level clusters indicated that 33.1% screened positive for probable depression, 28.2% for probable anxiety and 31.2% for probable bipolar/mania. These categories were not mutually exclusive; some participants screened positive for more than one cluster.

### 3.4. Bivariate Associations

Table 3 presents associations between psychological distress and demographic, socioeconomic and lifestyle factors. Psychological distress was significantly more prevalent among females (76.7% vs. 67.5% in males) and among students aged 16 and 17 years compared with those aged 15 or 18 years. Lower household income (<5000 rials) was associated with the highest distress prevalence (85.1%), whereas students from families earning > 15,000 rials had the lowest prevalence (56.2%). Distress prevalence was strongly related to sleep duration; students sleeping less than 4 h per night had a prevalence of 90.9% compared with 58.2% among those sleeping 7–9 h. Physical activity was also associated with distress: students reporting no activity had the highest prevalence (82.3%). Bullying exposure had the largest effect, with 89.9% of students who reported verbal or psychological bullying screening positive for distress. School type, parental education, number of siblings and history of mental conditions were not significantly associated with distress.

Across all three outcomes—probable depression, anxiety and bipolar/mania—a few factors emerged as consistently important. Girls were significantly more likely than boys to screen positive for anxiety and bipolar/mania and showed a slightly higher rate of depressive symptoms. Students attending government schools had higher prevalences of anxiety and bipolar/mania than those in private schools. Socioeconomic disadvantage increased risk: adolescents from low-income households and those whose fathers had little formal education were much more likely to report symptoms of anxiety and bipolar/mania, whereas income gradients for depression were less pronounced.

Sleep patterns were strongly linked to mental health status; students sleeping the recommended seven to nine hours each night had the lowest rates of all three outcomes, while those sleeping less than four hours or more than nine hours had markedly higher rates of depression, anxiety and bipolar/mania. Similarly, physical inactivity was associated with greater psychological distress across the board; the highest prevalences were observed among students who reported no regular exercise. Finally, bullying was one of the most potent risk factors: those exposed to verbal, psychological or physical bullying were several times more likely to meet the thresholds for depression, anxiety and bipolar/mania compared with peers who were not bullied. Age, number of siblings, and maternal education showed little or no association with these mental health outcomes. Together, these patterns highlight the combined influence of gender, school environment, socioeconomic status, sleep, lack of physical activity and peer victimisation on adolescent mental health in Makkah.

### 3.5. Multivariable Logistic Regression

**Figure 1:**
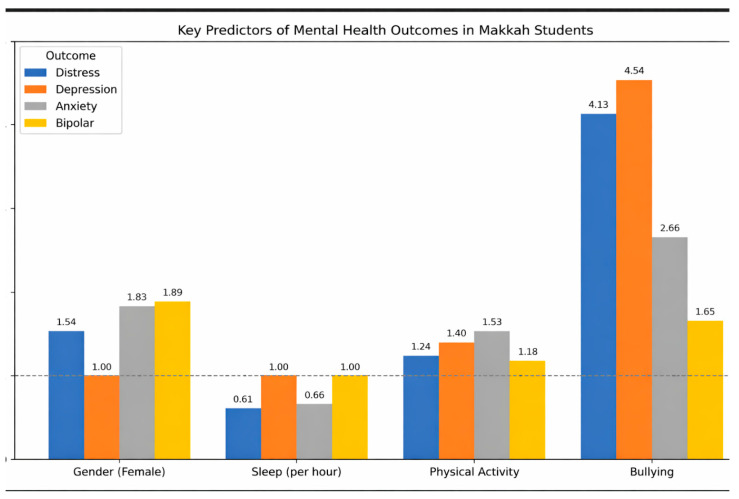
Illustrates the adjusted odds ratios for key predictors across all mental health outcomes. Dashed line means at 1 OR, there is no association of predictors across mental health outcomes.

Multivariable logistic regression models are presented in Table 4, Table 5, Table 6 and Table 7. Figure 1 summarizes the adjusted odds ratios for the key predictors across psychological distress and the three exploratory symptom groupings. In the model for psychological distress (Table 4), female gender remained a significant predictor (adjusted odds ratio [aOR] = 1.54; 95% confidence interval [CI] 1.01–2.60). Longer sleep duration was protective (aOR = 0.61 per additional hour; 95% CI 0.47–0.80). Lack of physical activity showed a modest positive association with distress (aOR = 1.24; 95% CI 1.04–1.48), and bullying exposure was the strongest predictor (aOR = 4.13; 95% CI 2.19–7.78). Other factors, including age, academic year, school type, income, parental education, number of siblings and medication use, were not significant.

In the model for probable depression (Table 5), a history of psychological or mental conditions substantially increased the odds of depression (aOR = 11.56; 95% CI 1.99–134.45), while current medication use was associated with reduced odds (aOR = 0.55; 95% CI 0.31–0.98). Lack of physical activity remained positively associated with depression (aOR = 1.40; 95% CI 1.18–1.66), and bullying exposure increased the odds more than four-fold (aOR = 4.54; 95% CI 2.88–7.17). Sleep duration was not a significant predictor after adjustment.

In the model for probable anxiety (Table 6), older age was protective (aOR = 0.69; 95% CI 0.47–1.00). Female gender (aOR = 1.83; 95% CI 1.10–3.05) and higher academic year (aOR = 1.68; 95% CI 1.07–2.64) increased the odds of anxiety. Medication use (aOR = 0.45; 95% CI 0.25–0.81) and higher father’s education (aOR = 0.80; 95% CI 0.65–0.98) were protective. Longer sleep duration reduced the odds (aOR = 0.66; 95% CI 0.51–0.87). Lack of physical activity (aOR = 1.53; 95% CI 1.29–1.83) and bullying (aOR = 2.66; 95% CI 1.65–4.29) were associated with higher odds of anxiety.

In the model for probable bipolar/mania (Table 7), female gender (aOR = 1.89; 95% CI 1.19–3.00), lack of physical activity (aOR = 1.18; 95% CI 1.01–1.39) and bullying exposure (aOR = 1.65; 95% CI 1.04–2.60) increased the odds of bipolar/mania. Medication use was associated with lower odds (aOR = 0.54; 95% CI 0.31–0.93). Other variables were not significant.

## 4. Discussion

This cross-sectional study provides a comprehensive assessment of psychological distress and probable depression, anxiety and bipolar/mania symptoms among high-school students in Makkah. Using the Arabic GHQ-30, more than two-thirds of the sample screened positive for psychological distress and roughly one-third screened positive for probable depression or anxiety. These estimates exceed global adolescent mental health prevalence figures; the World Health Organization reports that about one in seven adolescents experiences a mental disorder. Regional studies using GHQ-based screening tools have also documented substantial levels of psychological distress among adolescents. For example, an Iranian high-school study using the GHQ-28 reported symptoms of anxiety, depression, and social impairment in 40%, 33%, and 32% of students, respectively, while another Iranian study using the GHQ-12 found that 35.4% of students scored above the threshold for probable psychological distress [1,26,27]. Saudi studies have likewise documented elevated levels of depressive, anxiety, and related psychological symptoms among secondary-school students [12,13,24,28]. Our exploratory analysis suggests that predefined GHQ-30 item groupings may help describe symptom-pattern distributions and correlates in this population; however, because these groupings have not been formally validated, they should be interpreted as exploratory rather than diagnostic. Our findings therefore highlight a substantial mental health burden among students in Makkah.

Consistent with previous research, female students were more likely than males to experience psychological distress and to screen positive for anxiety or bipolar/mania. Large meta-analyses and epidemiological surveys indicate that gender differences in depression and anxiety emerge in early adolescence and persist across cultures [29]. Biological factors (such as hormonal changes) and psychosocial factors (including gender-specific stress and expectations) likely contribute to these differences.

Sleep duration emerged as a robust protective factor. Students sleeping fewer than 4 h per night had markedly higher prevalence of distress and symptom clusters, whereas sleep durations of 7–9 h were associated with substantially lower odds of psychological symptoms. These findings align with evidence that insufficient sleep significantly increases emotional dysregulation and vulnerability to psychiatric symptoms [30,31]. Sleep deprivation disrupts prefrontal–amygdala connectivity, leading to heightened emotional reactivity and impaired cognitive control [32]. Promoting healthy sleep habits may therefore be an effective strategy for improving adolescent mental health.

Bullying exposure was among the strongest predictors of psychological distress and all symptom clusters. Students subjected to verbal, psychological or combined bullying had dramatically higher odds of distress, depression, anxiety and bipolar/mania. This is supported by systematic reviews showing that bullying victimisation is consistently associated with depression, anxiety and suicidal ideation [33]. The Health Behaviour in School-aged Children (HBSC) international survey confirms that bullying victimisation contributes to psychosomatic and psychological symptoms in adolescents [34]. Studies in the Gulf region, including Saudi Arabia and the United Arab Emirates, report similar patterns of increased psychological symptoms among bullied adolescents [35,36]. Broader school-based adolescent research has likewise shown that psychosocial distress is linked to social adversity and violence-related exposures, including bullying, physical attacks, fighting, and food insecurity, whereas supportive peer relationships may be protective [37]. Effective anti-bullying programmes and supportive school environments are therefore essential components of mental health promotion.

Bullying and female gender emerged as the strongest risk factors for distress, depression, anxiety and bipolar/mania, whereas sufficient sleep had a marked protective effect. Physical inactivity was also associated with increased risk across outcomes.

The positive association between lack of physical activity and mental health problems observed in the multivariable models is consistent with the global literature: insufficient activity is known to increase the risk of mental-health problems. Most literature indicates that regular, moderate physical activity improves mood and reduces symptoms of depression and anxiety [38,39].

Lower socioeconomic status was associated with higher distress and symptom prevalence in descriptive analyses, consistent with evidence that economic disadvantage exacerbates psychological vulnerability through chronic stress, reduced access to supportive environments and increased exposure to adversity [40]. Income did not remain an independent predictor after adjustment, suggesting its effects may operate through mediators such as sleep, bullying or school environment. A history of mental health conditions strongly predicted probable depression, while medication use appeared protective across several models. These findings underscore the importance of early identification and treatment; prospective studies show that untreated childhood psychiatric problems are associated with adverse adult outcomes [41].

Academic performance, number of siblings and parental education were not consistently associated with mental health outcomes. Previous research reports mixed findings regarding these factors; some studies suggest that academic achievement and parental education are protective, whereas others report minimal or context-dependent effects [42]. In this study, high overall academic performance and a predominance of university-educated parents may have limited variability and statistical power.

Overall, the determinants of mental health observed in Makkah—female gender, inadequate sleep, bullying, socioeconomic disadvantage and mental health history—mirror those documented in international studies. The high overall burden of psychological symptoms indicates the need for school-based mental health programmes, improved sleep-hygiene education, robust anti-bullying initiatives and early screening using validated tools such as the GHQ-30.

A number of limitations should be acknowledged. First, because this was a cross-sectional survey, it provides only a single snapshot and cannot establish temporal relationships or infer causation. Associations observed between sleep, lack of physical activity, bullying and mental health outcomes could be bidirectional, and longitudinal studies would be needed to clarify causal pathways. Second, the study population comprised only high-school students from four schools in Makkah; although stratified random sampling was used, the findings may not generalize to adolescents in other regions or to out-of-school youth. Self-administered questionnaires are also subject to recall errors and social desirability bias; stigma surrounding mental health may have led some students to under-report symptoms or experiences such as bullying. Third, the General Health Questionnaire-30 is a screening tool for general psychological distress, not a diagnostic instrument; it may not detect severe or chronic psychiatric disorders and can be influenced by cultural and linguistic factors. The subscale groupings used here (for depression, anxiety and bipolar-like symptoms) have not been formally validated and should be interpreted cautiously. Although related Arabic and regional GHQ instruments have shown acceptable reliability and validity in other populations, these studies involved different GHQ versions and settings; therefore, adolescent-specific psychometric validation of the Arabic GHQ-30 and of the exploratory item clusters used here remains important [43,44]. Fourth, although we recruited additional participants to compensate for potential non-response, as recommended by sample-size guides, the final analytic sample was smaller than the target and some subgroups (e.g., students with a history of mental illness or taking medications) were small. This limited the precision of estimates and produced wide confidence intervals for some predictors. In addition, although the study used a stratified two-stage cluster sampling design and clustering was present at the school and class levels, the final analyses did not use a formal complex-samples framework and the regression models did not include random effects. Therefore, the precision of the estimated associations should be interpreted with caution, as clustering-related variation may not have been fully accounted for. Finally, unmeasured factors such as trauma exposure, substance use, or family history may confound the observed relationships; the cross-sectional design is particularly vulnerable to residual confounding and selection bias.

## 5. Conclusions and Recommendations

This study reveals a considerable burden of psychological distress and probable depression, anxiety and bipolar/mania symptoms among high-school students in Makkah. Key determinants include female gender, inadequate sleep, bullying exposure and lack of physical activity. Socioeconomic disadvantage and a history of mental health conditions also contribute to vulnerability. These findings underscore the need to recognise mental health as a core component of adolescent well-being, particularly within school environments where academic pressures, social transitions and lifestyle changes can heighten vulnerability.

Schools should integrate routine mental health screening programmes using validated tools such as the GHQ-30, alongside sleep-hygiene education and comprehensive anti-bullying initiatives. Teachers and school counsellors require training to recognise mental health problems and to provide appropriate support or referrals. Expanding access to school-based counselling services and creating confidential, youth-friendly mental health clinics within or near schools could encourage help-seeking behaviours [22]. Periodic school-based surveillance may also complement one-time screening, as international survey frameworks have been used to monitor adolescent behavioural risk and protective factors and to inform school health policies at country level [45,46]. Public-health authorities should implement awareness campaigns to reduce stigma and encourage family involvement in supporting adolescents. Future research should investigate and further validate GHQ-30 item clusters as screening tools for specific disorders.

## Figures and Tables

**Table 1:** Demographic characteristics of the study students, Makkah, Saudi Arabia (N = 535).

Socio-Demographic Data	No	%
**Age in years**		
15	51	9.5%
16	130	24.3%
17	245	45.8%
18	109	20.4%
**Gender**		
Male	363	67.9%
Female	172	32.1%
**The study year**		
1st secondary school year	96	17.9%
2nd secondary school year	164	30.7%
3rd secondary school year	275	51.4%
**School type**		
Governmental	366	68.4%
Private	169	31.6%
**Do you have a history of psychological/mental conditions?**		
Yes	6	1.1%
No	529	98.9%
**Do you take any medications regularly?**		
Yes, neurological or psychiatric medications	4	.7%
Yes, medications for other diseases	55	10.3%
None	476	89.0%
**Monthly income**		
<5000 SR	67	12.5%
5000–10,000 SR	76	14.2%
10,000–15,000 SR	40	7.5%
>15,000 SR	89	16.6%
I prefer not to answer	263	49.2%
**Father education**		
Illiterate	13	2.4%
Primary education	20	3.7%
Preparatory education	42	7.9%
Secondary education	136	25.4%
University education	213	39.8%
Post-graduate degree	111	20.7%
**Mother education**		
Illiterate	23	4.3%
Primary education	23	4.3%
Preparatory education	65	12.1%
Secondary education	137	25.6%
University education	236	44.1%
Post-graduate degree	51	9.5%
**Number of brothers and sisters**		
None	8	1.5%
1–2	92	17.2%
3–4	224	41.9%
5+	211	39.4%

**Table 2:** Lifestyle Habits, bullying exposure, and academic performance of high-school students in Makkah (N = 535).

Items	No	%
**Daily sleep hours**		
<4 h/day	33	6.2%
4–6 h/day	242	45.2%
7–9 h/day	201	37.6%
>9 h/day	59	11%
**Physical activity level**		
<60 min per week	143	26.7%
60–400 min per week	158	29.5%
>400 min per week	59	11%
I do not engage in any physical activity	175	32.7%
**Have you been subjected to any kind of bullying from your schoolmates during the past three years?**		
I have not been subjected to any type of bullying.	415	77.6%
Yes, I have been subjected to verbal or psychological bullying.	109	20.4%
Yes, I was subjected to physical and psychological bullying.	7	1.3%
Yes, I was subjected to physical bullying.	4	0.7%
**Academic performance**		
Range	20–100	
Mean ± SD	94.5 ± 8.0	

**Table 3:** Factors associated with high-school students’ psychological distress in Makkah.

Factors		Psychological Distress				*p*-Value
		No		Yes		
		No	%	No	%	
Age in years	15	26	51.0%	25	49.0%	0.002 *
	16	37	28.5%	93	71.5%	
	17	60	24.5%	185	75.5%	
	18	35	32.1%	74	67.9%	
Gender	Male	118	32.5%	245	67.5%	0.028 *
	Female	40	23.3%	132	76.7%	
School type	Governmental	99	27.0%	267	73.0%	0.064
	Private	59	34.9%	110	65.1%	
Do you have a history of psychological/mental conditions?	Yes	1	16.7%	5	83.3%	0.487 ^
	No	157	29.7%	372	70.3%	
Monthly income	<5000 SR	10	14.9%	57	85.1%	0.001 *
	5000–10,000 SR	23	30.3%	53	69.7%	
	10,000–15,000 SR	15	37.5%	25	62.5%	
	>15,000 SR	39	43.8%	50	56.2%	
	I prefer not to answer	71	27.0%	192	73.0%	
Father education	Illiterate	2	15.4%	11	84.6%	0.652
	Primary education	5	25.0%	15	75.0%	
	Preparatory education	10	23.8%	32	76.2%	
	Secondary education	43	31.6%	93	68.4%	
	University education	68	31.9%	145	68.1%	
	Post-graduate degree	30	27.0%	81	73.0%	
Mother education	Illiterate	6	26.1%	17	73.9%	0.525
	Primary education	7	30.4%	16	69.6%	
	Preparatory education	19	29.2%	46	70.8%	
	Secondary education	38	27.7%	99	72.3%	
	University education	78	33.1%	158	66.9%	
	Post-graduate degree	10	19.6%	41	80.4%	
Number of brothers and sisters	None	2	25.0%	6	75.0%	0.906
	1–2	26	28.3%	66	71.7%	
	3–4	64	28.6%	160	71.4%	
	5+	66	31.3%	145	68.7%	
Daily sleep hours	<4 h/day	3	9.1%	30	90.9%	0.001 *
	4–6 h/day	54	22.3%	188	77.7%	
	7–9 h/day	84	41.8%	117	58.2%	
	>9 h/day	17	28.8%	42	71.2%	
Physical activity level	<60 min per week	44	30.8%	99	69.2%	0.001 *
	60–400 min per week	63	39.9%	95	60.1%	
	>400 min per week	20	33.9%	39	66.1%	
	I do not engage in any physical activity	31	17.7%	144	82.3%	
Have you been subjected to any bullying from your schoolmates during the past three years?	I have not been subjected to any bullying.	145	34.9%	270	65.1%	0.001 *^
	Yes, I have been subjected to verbal or psychological bullying.	11	10.1%	98	89.9%	
	Yes, I was subjected to physical and psychological bullying.	1	14.3%	6	85.7%	
	Yes, I was subjected to physical bullying.	1	25.0%	3	75.0%	

* *p* < 0.05 (statistically significant); ^ Fisher’s exact test was used when expected cell counts were small.

**Table 4:** Multivariate logistic regression of predictors for psychological distress among secondary school students in Makkah.

Predictors	*p*-Value	ORA	95% CI	
			Lower	Upper
Age in years	0.485	1.14	0.79	1.62
Female vs. Male gender	0.049 *	1.54	1.01	2.60
Higher academic year	0.444	1.18	0.77	1.79
Private school vs. Governmental	0.924	0.97	0.57	1.65
History of psychological/mental conditions	0.771	1.41	0.14	14.50
Receive any medications	0.198	0.63	0.31	1.27
Higher family income	0.841	0.98	0.85	1.15
Higher father’s education	0.859	1.02	0.82	1.26
Higher mother’s education	0.464	1.07	0.89	1.29
Number of brothers/sisters	0.545	0.92	0.70	1.21
Academic performance (high grades)	0.202	0.98	0.94	1.01
Daily sleep hours	0.001 *	0.61	0.47	0.80
Physical activity engagement per week	0.015 *	1.24	1.04	1.48
Subjected to any kind of bullying from your schoolmates	0.001 *	4.13	2.19	7.78

ORA: Adjusted odds ratio; CI: Confidence Interval; * *p* < 0.05 (significant).

**Table 5:** Multivariate logistic regression of predictors for depression among secondary school students in Makkah.

Predictors	*p*-Value	ORA	95% CI	
			Lower	Upper
Age in years	0.547	0.90	0.63	1.28
Female vs. Male gender	0.768	0.93	0.57	1.52
Higher academic year	0.388	1.20	0.79	1.82
Private school vs. Governmental	0.132	0.67	0.39	1.13
History of psychological/mental conditions	0.048 *	11.56	1.99	134.45
Receive any medications	0.041 *	0.55	0.31	0.98
Higher family income	0.199	1.10	0.95	1.27
Higher father’s education	0.286	0.90	0.73	1.10
Higher mother’s education	0.996	1.00	0.83	1.20
Number of brothers/sisters	0.934	1.01	0.78	1.32
Academic performance (high grades)	0.501	1.01	0.98	1.04
Daily sleep hours	0.111	.81	0.63	1.05
Physical activity engagement per week	0.001 *	1.40	1.18	1.66
Subjected to any kind of bullying from your schoolmates	0.001 *	4.54	2.88	7.17

ORA: Adjusted odds ratio; CI: Confidence Interval; * *p* < 0.05 (significant).

**Table 6:** Multivariate Logistic Regression of Predictors for Anxiety Among Secondary School Students in Makkah.

Predictors	*p*-Value	ORA	95% CI	
			Lower	Upper
Age in years	0.047 *	0.69	0.47	1.00
Female vs. Male gender	0.019 *	1.83	1.10	3.05
Higher academic year	0.024 *	1.68	1.07	2.64
Private school vs. Governmental	0.816	0.93	0.52	1.67
History of psychological/mental conditions	0.185	3.80	0.53	27.38
Receive any medications	0.007 *	0.45	0.25	0.81
Higher family income	0.823	0.98	0.85	1.14
Higher father’s education	0.032 *	0.80	0.65	0.98
Higher mother’s education	0.625	0.95	0.78	1.16
Number of brothers/sisters	0.852	1.03	0.77	1.36
Academic performance (high grades)	0.743	1.00	0.97	1.02
Daily sleep hours	0.003 *	0.66	0.51	0.87
Physical activity engagement per week	0.001 *	1.53	1.29	1.83
Subjected to any kind of bullying from your schoolmates	0.001 *	2.66	1.65	4.29

ORA: Adjusted odds ratio; CI: Confidence Interval; * *p* < 0.05 (significant).

**Table 7:** Multivariate logistic regression of predictors for bipolar/mania among secondary school students in Makkah.

Predictors	*p*-Value	ORA	95% CI	
			Lower	Upper
Age in years	0.700	0.93	0.66	1.33
Female vs. Male gender	0.007 *	1.89	1.19	3.00
Higher academic year	0.406	1.19	0.79	1.79
Private school vs. Governmental	0.070	0.60	0.35	1.04
History of psychological/mental conditions	0.928	0.92	0.16	5.20
Receive any medications	0.026 *	0.54	0.31	0.93
Higher family income	0.770	0.98	0.85	1.12
Higher father’s education	0.373	0.91	0.75	1.11
Higher mother’s education	0.815	1.02	0.85	1.23
Number of brothers/sisters	0.820	0.97	0.75	1.26
Academic performance (high grades)	0.313	1.01	0.99	1.04
Daily sleep hours	0.066	0.79	0.62	1.02
Physical activity engagement per week	0.040 *	1.18	1.01	1.39
Subjected to any kind of bullying from your schoolmates	0.033 *	1.65	1.04	2.60

ORA: Adjusted odds ratio; CI: Confidence Interval; * *p* < 0.05 (significant).

## Data Availability

The data are not publicly available because they contain sensitive information from minors and are subject to ethical and institutional restrictions. De-identified data may be available from the corresponding author on reasonable request and with permission from the relevant ethics authority.

## Supplementary Materials

The following supporting information can be downloaded at: https://www.mdpi.com/article/10.3390/ijerph23060733/s1, Supplementary File S1: General health questionnaire.
