# Supplementary material for: Psychological Distress and Associated Factors Among High-School Students in Makkah, Saudi Arabia: A Cross-Sectional Study Using the Arabic GHQ-30
Source: Int J Environ Res Public Health. 2026 May 30;23(6):733. doi: 10.3390/ijerph23060733 (PMC13299696; doi:10.3390/ijerph23060733)
Supplement: Supplementary file 1 [file ijerph-23-00733-s001.zip › ijerph-4299755-supplementary.pdf]

GENERAL HEALTH QUESTIONNAIRE

R SERIAL NO. :IDNO

INTERVIEWER NO.

	**GHQ-30**	

Please read this carefully:

We should like to know if you have any medical complaints, and how your health has been in general, *over the past few weeks.* Please answer ALL the questions on the following pages simply by under-lining the answer which you think most nearly applies to you. Remember that we want to know about present and recent complaints, not those that you had in the past.

It is important that you try to answer ALL the questions.

Thank you very much for your co-operation.

HAVE YOU RECENTLY:

GHQ1.A	1.	been able to concentrate on whatever you're doing?	Better than usual	Same as usual	Less than usual	Much less than usual
GHQ2.A	2.	lost much sleep over worry?	Not at all	No more than usual	Rather more than usual	Much more than usual
GHQ3.A	3.	been having restless, disturbed nights?	Not at all	No more than usual	Rather more than usual	Much more than usual
GHQ4.A	4.	been managing to keep yourself busy and occupied?	More so than usual	Same as usual	Rather less than usual	Much less than usual
GHQ5.A	5.	been getting out of the house as much as usual?	More so than usual	Same as usual	Less than usual	Much less than usual
GHQ6.A	6.	been managing as well as most people would in your shoes?	Better than most	About the same	Rather less well	Much less well
GHQ7.A	7.	felt on the whole you were doing things well	Better than usual	About the same	Less well than usual	Much less well
GHQ8.A	8.	been satisfied with the way you've carried out your task?	More satisfied	About same as usual	Less satisfied than usual	Much less satisfied
GHQ9.A	9.	been able to feel warmth and affection for those near to you?	Better than usual	About same as usual	Less well than usual	Much less well
GHQ10.A	10.	been finding it easy to get on with other people?	Better than usual	About same as usual	Less well than usual	Much less well
GHQ10.A	11.	spent much time chatting with people?	More time than usual	About same as usual	Less time than usual	Much less than usual
GHQ12.A	12.	felt that you are playing a useful part	More so	Same	Less useful	Much less
GHQ13.A	13.	felt capable of making decisions about things?	More so than usual	Same as usual	Less so than usual	Much less capable

HAVE YOU RECENTLY:

GHQ14.A	14.	felt constantly under strain?	Not at all	No more than usual	Rather more than usual	Much more than usual
GHQ15.A	15.	felt you couldn't overcome your difficulties?	Not at all	No more than usual	Rather more than usual	Much more than usual
GHQ16.A	16.	been finding life a struggle all the time?	Not at all	No more than usual	Rather more than usual	Much more than usual
GHQ17.A	17.	been able to enjoy your normal day-to-day activities?	More so than usual	Same as usual	Less so than usual	Much less than usual
GHQ18.A	18.	been taking things hard?	Not at all	No more than usual	Rather more than usual	Much more than usual
GHQ19.A	19.	been getting scared or panicky for no good reason?	Not at all	No more than usual	Rather more than usual	Much more than usual
GHQ20.A	20.	been able to face up to your problems?	More so than usual	Same as usual	Less able than usual	Much less able
GHQ21.A	21.	found everything getting on top of you?	Not at all	No more than usual	Rather more than usual	Much more than usual
GHQ22.A	22.	been feeling unhappy and depressed?	Not at all	No more than usual	Rather more than usual	Much more than usual
GHQ23.A	23.	been losing confidence in yourself?	Not at all	No more than usual	Rather more than usual	Much more than usual
GHQ24.A	24.	been thinking of yourself as a worthless person?	Not at all	No more than usual	Rather more than usual	Much more than usual
GHQ25.A	25.	felt that life is entirely hopeless?	Not at all	No more than usual	Rather more than usual	Much more than usual
GHQ26.A	26.	been feeling hopeful about your own future?	More so than usual	About same as usual	Less so than usual	Much less hopeful
GHQ27.A	27.	been feeling reasonably happy, all things considered?	More so than usual	About same as usual	Less so than usual	Much less than usual
GHQ28.A	28.	been feeling nervous and strung-up all the time?	Not at all	No more than usual	Rather more than usual	Much more than usual
GHQ29.A	29.	felt that life isn't worth living?	Not at all	No more than usual	Rather more than usual	Much more than usual
GHQ30.A	30.	found at times you couldn't do anything because your nerves were too bad?	Not at all	No more than usual	Rather more than usual	Much more than usual

Copyright © David Goldberg, 1978
Published by The NFER-NELSON Publishing Company,
Darville House, 2 Oxford Road East, Windsor, SL4 1DF, Berks.
All rights reserved. This work may not be reproduced by any means, even within the terms of a Photocopying Licence, without the written permission of the publisher.

1 (4.85)

Printed in Great Britain

code: 4075 03 4
